# The complete mitochondrial genome of the catfish *Silurus cochinchinensis* (Siluriformes: Siluridae)

**DOI:** 10.1080/23802359.2018.1495121

**Published:** 2018-10-25

**Authors:** Xiangchen Ye, Lingjing Wei, Yejian Lv, Baojiang Gan, Xijun Gan, Zhongzuo Teng

**Affiliations:** Aquatic Species Introduction and Breeding Center of Guangxi, Nanning, China

**Keywords:** Mitochondrial genome, *Silurus cochinchinensis*, structural organization

## Abstract

In this study, the complete mitochondrial genome of *Silurus cochinchinensis* was reported to be 16,501 bp in length, consisting of 13 protein-coding genes, 2 ribosomal RNA genes, 22 transfer RNA genes, and one control region. The structural organization and gene order were equivalent to other bony fishes. The complete genome of *S. cochinchinensis* would provide the basic dataset for further research on phylogenetics and conservation genetics of catfishes.

*Silurus cochinchinensis* belonging to Siluridae family is often found in slow-flowed mountain stream or cave (Kobayakawa 1989; Guangxi Academy of Fishery Sciences 2006). *S. cochinchinsis* widely distributes in Thailand, Burma, Vietnam, and South China (Kobayakawa 1989; Wang 2011). Though it generates low productivity and economic value, this small bottom-dwelling fish is the perfect materials for research the affinity of freshwater fish in South China (Wang 2011; Xu et al. 2015). Here, the whole genome of *S. cochinchinensis* was reported for the first time using polymerase chain reaction.

The sample of *S. cochinchinensis* was collected from a stream of Lingshan county (22°24′N, 109°17′E), Guangxi Province, China. Its caudal fin was preserved in anhydrous alcohol and total DNA was extracted using traditional phenol/chloroform method. Both of the specimen and the DNA samples were stored in Aquatic species introduction and breeding center of Guangxi. Seventeen pairs of primers were designed to amplify contiguous fragments of the entire mtDNA of *S. cochinchinensis*, with each segment overlapping the next more than 100 bp. The mtDNA sequences data were analyzed and assembled using DNAstar Lasergene 7.0 software. The locations of protein-coding and rRNA genes were identified by comparison with other previously-published mitochondrial genome sequences of bony fishes on the BLAST (http://blast.ncbi.nlm.nih.gov/BLAST/). tRNA genes were determined by sequence homology and their secondary structure (Kumazawa and Nishida, 1993).

The mitochondrial genome of *S. cochinchinensis* was 16,501 bp in length and has been deposited in Genbank with accession number MH202954. The structural organization and gene order of the mtDNA was identical to other typical bony fishes, which contains the following: 13 protein-coding genes, 2 ribosomal RNAs, 22 transfer RNAs, and one control region (Ng and Freyhof 2001; Xu et al. 2015). In the overall, base composition of the *S. cochinchinensis* mtDNA genome was estimated to be 31.53% A, 14.68% G, 25.43% T, and 28.36% C, with a slight A + T bias (56.96%). Most of the protein-coding genes started with ATG except CO1, which started with GTG. Eight protein-coding genes (ND1, CO1, ATPase8, ATPase6, CO3, ND4L, ND5, and ND6) genes were terminated with TAA, ND2 and ND3 genes were both ended with TAG, and CO2, ND4, and cytb genes were ended with incomplete stop codons of T. The length of 22 tRNAs were ranged from 67 bp to 74 bp, which separated ribosome RNAs, protein-coding genes, and D-loop. The 12S and 16S ribosomal RNA genes were flanked by tRNA^Val^ with 949 bp and 1669 bp, respectively. The control region was flanked by the tRNA^pro^ and tRNA^phe^ genes with 880 bp in length, which was shorter than other closely related species.

To validate the phylogenetic position of *S. cochinchinensis*, MEGA7.0 software was used to construct a Neighbor-joining tree containing complete mitogenetic sequences of 12 other bony fishes downloaded from GenBank (Figure 1). *Cyprinus carpio* was used as an outgroup member for tree rooting. In this tree, *S. cochinchinensis* is more closely related to *Pterocryptis cochinchinensis*, rather than to other *Silurus* species.

**Figure 1.**
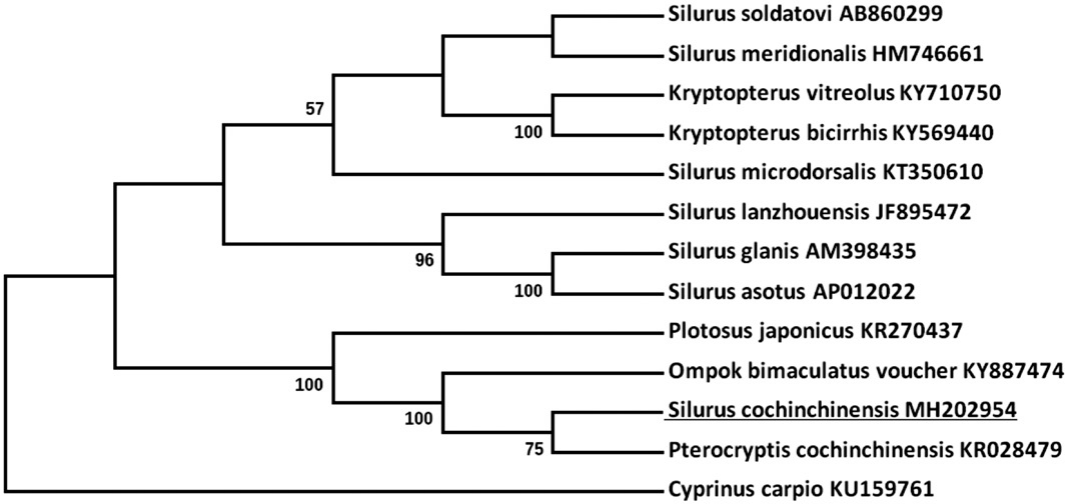
Molecular phylogeny of *Silurus cochinchinensis* and other teleost varieties based on complete mitogenome. The mtDNA sequences are downloaded from Genbank and the phylogenic tree is constructed using Neighbor-joining method with 1000 bootstrap replicates.
